# Maturation of B Cells in the Lamina Propria of Human Gut and Bronchi in the First Months of Human Life

**DOI:** 10.1155/1998/42138

**Published:** 1998

**Authors:** Jamal El Kaissouni, Marie C. Bene, Sophie Thionnois, Pierre Monin, Michel Vidailhet, Gilbert C. Faure

**Affiliations:** 1 Laboratoire d'Immunologie Faculté de Médecine BP 184 Vandoeuvre les Nancy 54500 France; 2 Hopital d'Enfants CHU de Brabois Vandoeuvre Les Nancy France

**Keywords:** Bronchi, gut, human, MALT, maturation, plasma cells

## Abstract

Little is known of the maturation of the mucosae-associated lymphoid tissue (MALT) in man, because, for ethical reasons, tissues from newborns are not easy to obtain. We used the opportunity provided by autopsies systematically performed in infants who died of Sudden Infant Death Syndrome (SIDS) to study the maturation of the MALT after birth. Gut and bronchus samples of 90 infants from postpartum to 90 months and who died from SIDS were collected and studied by histological and immunofluorescence examination. Plasma cells, absent at birth, appeared within a few hours after birth and initially were of the IgM isotype. IgA plasma cells appeared at 12 days. These cells were first observed in gut and later in bronchi, indicating that maturation of the gut precedes that of bronchi. The number of plasma cells increased rapidly over time and IgA plasma cells became predominant after 3 weeks in the gut and 6 weeks in bronchi. At birth, only small IgM bearing B-cell foci were seen and organized germinal centers appeared to develop over a few days, first in the gut and only later in bronchi. These results confirm that, in man, the MALT organization at birth is still in its fetal form and that maturation depends on intestinal challenges and evolves over several weeks before IgA becomes the predominant isotype secreted.


*Developmental Immunology*, 1998, Vol. 5, pp. 153–159
Reprints available directly from the publisher
Photocopying permitted by license only

© 1998 OPA (Overseas Publishers Association)
Amsterdam B.V. Published under license
under the Harwood Academic Publishers imprint,
part of the Gordon and Breach Publishing Group
Printed in Malaysia

### Maturation of B Cells in the Lamina Propria of Human Gut and Bronchi in the First Months of Human Life

JAMAL EL KAISSOUNI[a], MARIE C. BENE[a], SOPHIE THIONNOIS[b], PIERRE MONIN[b], MICHEL VIDAILHET[b] and GILBERT C. FAURE[a*]

[a]*Laboratoire d'Immunologie, Faculté de Médecine, BP 184, Vandoeuvre les Nancy, 54500, France*; [b]*Hopital d'Enfants, CHU de Brabois, Vandoeuvre Les Nancy, France*

(*Received 11 June 1996*; *In final form 5 December 1996*)

Little is known of the maturation of the mucosae-associated lymphoid tissue (MALT) in man, because, for ethical reasons, tissues from newborns are not easy to obtain. We used the opportunity provided by autopsies systematically performed in infants who died of Sudden Infant Death Syndrome (SIDS) to study the maturation of the MALT after birth. Gut and bronchus samples of 90 infants from postpartum to 90 months and who died from SIDS were collected and studied by histological and immunofluorescence examination. Plasma cells, absent at birth, appeared within a few hours after birth and initially were of the IgM isotype. IgA plasma cells appeared at 12 days. These cells were first observed in gut and later in bronchi, indicating that maturation of the gut precedes that of bronchi. The number of plasma cells increased rapidly over time and IgA plasma cells became predominant after 3 weeks in the gut and 6 weeks in bronchi. At birth, only small IgM bearing B-cell foci were seen and organized germinal centers appeared to develop over a few days, first in the gut and only later in bronchi. These results confirm that, in man, the MALT organization at birth is still in its fetal form and that maturation depends on intestinal challenges and evolves over several weeks before IgA becomes the predominant isotype secreted.

*Keywords:* Bronchi, gut, human, MALT, maturation, plasma cells

### INTRODUCTION

Mucosae-associated lymphoid tissue (MALT) is one of the most important protection barriers toward the environment (Picker and Siegelman, 1993). Significant progress has been made in understanding the physiology of this system. However, little is known about its maturation in man. This is mostly due because infant's mucosal tissues are not easy to obtain for ethical reasons. It is also difficult to extrapolate the organization and possible functions of newborn's MALT from those of a fetus or an adult (Spencer and MacDonald, 1990). In the first case, the fetus is protected from external challenge and the mucosa is not stimulated by antigens or mitogens. In adults, the MALT has been submitted to repeated and prolonged

*Corresponding author.

antigen stimulation, allowing it to achieve an effective organisation (Mestecky and Mc Ghee, 1987).

Passage from fetal to adult MALT can be more easily studied in animals. Thus, in rat, several workers have studied the postnatal development of Peyer's patches (PP), which includes several steps (Eikelenboom et al., 1979). First, lymphoid cells accumulate against the *tunica muscularis* and constitute the future PP. After 8 to 12 days, the development of interfollicular areas containing T cells occurs, whereas B-cell containing follicles develop within 12 to 18 days. At this time, no secondary follicle is observed. The latter do not appear until 18 days of life and the adult organization of PP is hence accomplished (Sminia et al., 1983; Chen et al., 1995). Other studies have been reported in mice (Gutman and Weissman, 1972; Friedberg and Weissman, 1974) and pig (Allen and Porter, 1977).

Here, we used the opportunity of the autopsy systematically performed in cases of Sudden Infant Death Syndrome (SIDS) (Proust et al., 1992) to investigate the characteristics of the developing MALT in the first weeks of human life. In most cases, such children appear to have been healthy and developing normally, and although little is known about the aetiology of SIDS, it does not seem to involve anomalies of the immune system. A protocol was designed to sample duodenal and bronchial tissues in the course of such autopsies. We used these samples to study the postnatal organization of MALT and the time of appearance of plasma cells of IgG, IgA, or IgM isotype.

### RESULTS

### Histology

Histological examination of the biopsies showed normal well-conserved tissue structures in spite of the delay between death and autopsy. Gut samples allowed observation of duodenal villi, some lymphoid nodules, and glandular structures. The latter were well-developed except in the baby of 2 min of life, where these glands were smaller compared to other biopsies. Bronchial tissues allowed us observation of cartilage rings, a lamina propria containing glandular foci and covered by a layer of secretory epithelial cells and occasional lymphoid nodules.

No germinal center was observed in the gut sample collected after 2 min of life. Only three small foci of 20 to 30 lymphocytes could be seen in this tissue. Slightly larger similar formations were seen in the child deceased after 12 hr of life. At 48 hr, dome-covered structures with lymphoid cells accumulation were observed. In all the other samples, organized lymphoid nodules with the structure of germinal centers were regularly observed (Figure 1A).

In bronchial tissue, no lymphoid structure was observed at 2 min of life and a single small lymphoid nodule was observed at 12 hr of life. The number of these nodules increased with time after 8 weeks and they were almost always present in the other samples.

### Immunofluorescence

In the gut, no plasma cells were observed at 2 min of life, and the three small lymphoid foci appeared to be composed of B cells with surface IgM (Figure 1B). The first IgM plasma cells (20/mm$^2$) were observed at 2 days of life, and no IgA plasma cells were observed at that time. The number of these cells increased with time to reach 180 IgM plasma cells per mm$^2$ and the first IgA plasma cells were seen at 12 days of life. They were very few (20 to 40/mm$^2$), but their number increased in other specimens to reach 400 to 600 cells/mm$^2$ and further stabilized at this level (Figure 1C).

In early samples, IgM plasma cells were predominant, however, the kinetics of IgA plasma cells development appeared faster than that of IgM plasma cells, and within 3 weeks, IgA plasma cells became the predominant isotype. IgG plasma cells were seen after 4 weeks, but were in much smaller numbers than IgA or IgM plasma cells and remained at low levels in all later samples. The secretory component was brightly expressed by the epithelial cells of Lieberkühn glands and in all samples.

In bronchial tissue, a few scarce IgM-bearing B cells were observed at 2 min of life. They were slightly more numerous in the child who died after 12 hr. Germinal centers with a clear differentiation of the follicle and mantle zones, labeled with anti-IgM antiserum, were seen in all other samples.

The first IgA plasma cells appeared at 4 weeks. They were in low numbers (160/mm$^2$) similar to those of IgM plasma cells. The numbers of IgM and IgA plasma cells increased with time, but the kinetics was faster for IgA than for IgM plasma cells and IgA plasma cells became predominant by week 6 (Figure 1D). IgG plasma cells were first seen at 13 weeks and remained at very low levels (20/mm$^2$).

Secretory component was always found expressed brightly by overlying and glandular epithelial cells.

Figure 2 shows the kinetics of IgA and IgM plasma cells populations in gut and bronchi. It shows that intestinal plasma cells appear earlier than bronchial plasma cells. It also shows that IgA plasma cells develop later than IgM-producing cells.

### DISCUSSION

In this study, we approached the kinetics of postnatal maturation of two components of the MALT.

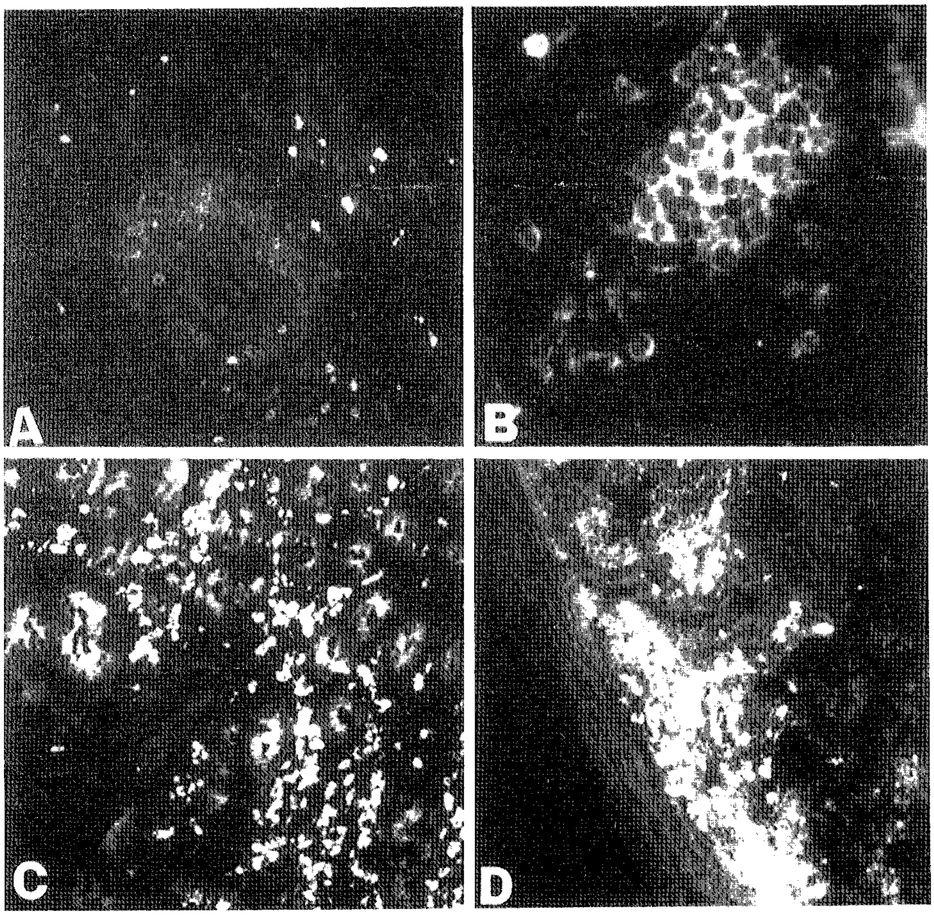

FIGURE 1 (A) Fully developed IgM+ germinal center in the gut of a child who died at 9 weeks of age. (Magnification × 100.) (B) Small nodule of IgM+ B cells in the gut of a newborn. (Magnification × 400.) (C) IgA plasma cells in the lamina propria of a gut sample at 20 weeks of age. (Magnification × 100.) (D) IgA plasma cells in the lamina propria of a bronchus sample at 20 weeks of age. (Magnification × 100.)

At birth, no plasma cell was observed either in gut or bronchial lamina propria. Only a few B lymphocytes were observed in the gut and later in bronchi, and no follicular structure was observed in the two tissues. This organization seems to represent that of fetal MALT, because this newborn was deceased 2 min after delivery. This very short time did not allow any modification of the mucosal immune system by any antigen stimulation. These findings are consistent with those of other authors who also reported that no plasma cell was seen at birth (Perkkio and Savilahti, 1980). This characteristic seems to be common to man and rat in which no plasma cells are present at birth (Sminia et al., 1983; Chen et al., 1995).

The fact that Ig-bearing cells appear first in gut tissue and later in bronchi indicates that maturation of the GALT occurs before that of bronchial tissue. This is also confirmed because the first plasma cells appeared in gut before bronchi. These results confirm the predominant role played by the GALT as an inductive site of the MALT. This notion of inductive and effector sites was first employed after the observation that cells from PP are able to repopulate the other lymphoid tissues in animals exposed to total body irradiation (Yednock and Rosen, 1989). Other experiences showed that oral antigen vaccination lead to the presence of antigen-specific producing cells first in peripheral blood before other mucosal lymphoid tissues (Czerkinsky et al., 1987). This study did not allow us to demonstrate directly that the B cells and plasma cells observed in bronchi are originating from the gut, however, the kinetics observed indicate

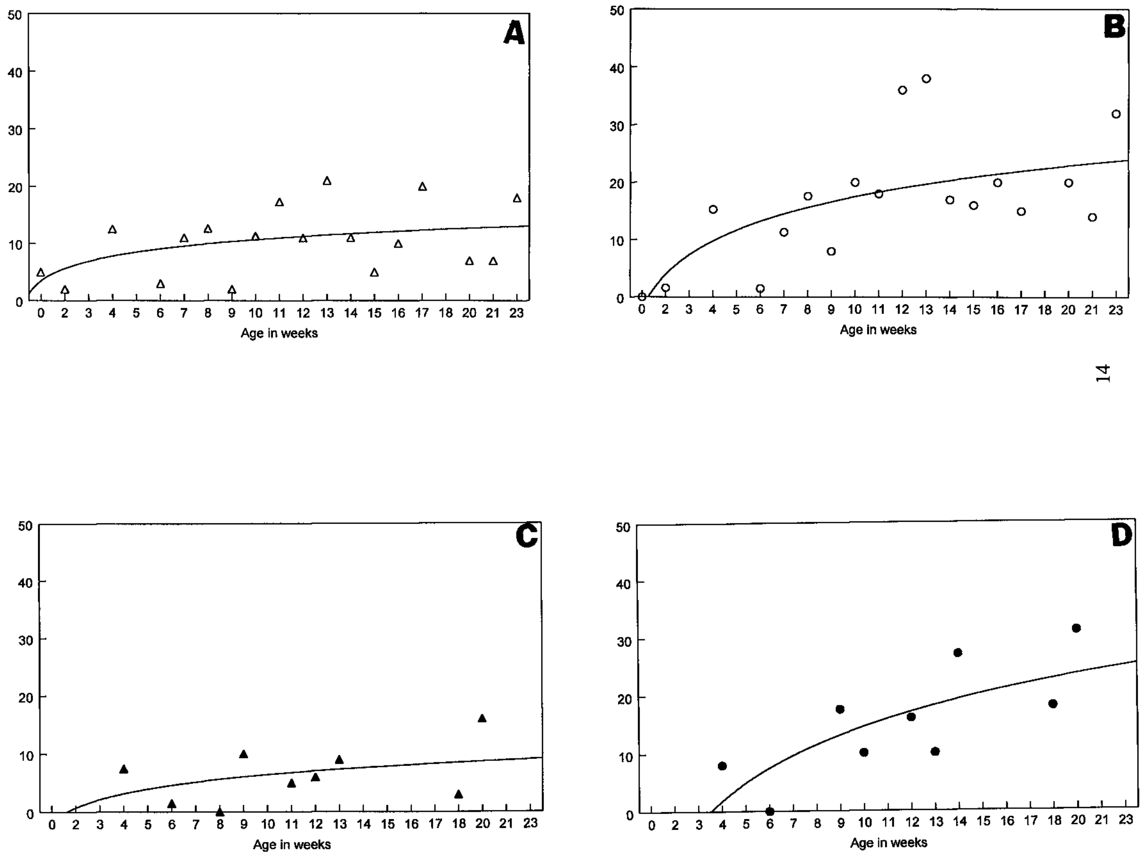

FIGURE 2 Time appearance of plasma cells in gut and bronchial mucosa. Data are expressed as mean numbers of plasma cells per field for each time point when tissue had been available. The curves are the computer-derived logarithmic fit for the experimental data. (A) IgM plasma cells in gut. (B) IgA plasma cells in gut. (C) IgM plasma cells in bronchi. (D) IgA plasma cells in bronchi.

that the maturation occurs primarily in gut before bronchi.

Savilahti studied the number of IgA plasma cells in human small intestine and in the rectal mucosa and reported that these cells are more numerous in the former (Savilahti, 1972). In our study, IgA and IgM were present at similar levels in gut and bronchi. This result suggests that even though GALT and bronchi are different in size, they harbor equal cell numbers per square $mm^2$. It suggests, too, that the bronchial lamina propria is an important component of the human's MALT even in the absence of organized BALT (Pabst, 1992).

Perkkio and Savilahti (1980) reported that no Ig-bearing cells nor plasma cells are present before 12 days after birth either in intestinal or rectal mucosae. Here, we demonstrated that B cells can be present at that time in the gut.

The first plasma cells observed were of IgM isotype and IgA plasma cells appeared at 12 days. This indicates that a new step was achieved in the way of MALT maturation and that appropriate signals had become available for isotype switch. This switching process is not random and is regulated by T cells and especially T helper cells (Snapper and Mond, 1993). An interaction between these two types of cells via the CD40 ligand is necessary for the switch to occur (Gascan et al., 1991). Cytokines also play a role in the switch because some of them preferentially induce switching to different isotypes (Edouard, 1993). The fact that plasma cells switched indicates (1) that the interaction of B and T cells via CD40/CD40L occurred, (2) that such interactions occur very soon after birth, and (3) indirectly, that T cells also matured rapidly after birth. This time appearance of plasma cells agrees with findings in rat where only few B cells are present at birth, whereas the first plasma cells appear after 12 days of life (Sminia et al., 1983).

The number of plasma cells regularly increased with time indicating cellular proliferation in response to antigen or mitogen stimulations. IgM plasma cells were initially predominant, however, and despite the fact that their number increased with time, IgA plasma cells increased more rapidly and this isotype became predominant in the two tissues. This confirms the important role of the microenvironment in determining isotype switching and B-cell proliferation.

The fact that no plasma cells were present in the mucosae of the newborn is consistent with the absence of immunoglobulins, either mucosal or systemic at birth (Taubman and Smith, 1993). This transient immune depression has no clinical incidence when passive immunity is provided by secretory IgA immunoglobulins contained in the mother's milk.

Torleiv and colleagues (1992) showed in tissular samples of the intestine that the cells observed in the lamina propria at 26 hr of life express HLA class II molecules without mentioning whether they were B cells, T cells, or dendritic cells. This suggests that functionally mature antigen-presenting cells are available at birth, able to present antigens to HLA II restricted CD4+ lymphocytes. It indirectly indicates that these cells are also able to process exogenous antigens. These results agree with our own findings in this study where we used some samples to study the expression of class II molecules by epithelial, stromal, and endothelial cells (data not shown). We found that the lymphocytes scattered in the lamina propria express class II molecules as early as at 2 min of life. This is consistent with findings of other authors who reported that class II molecules are expressed by either lymphocytes or dendritic cells in fetal gut lamina proria (Spencer et al., 1986). Class II molecules are normally expressed in antigen-presenting cells such as macrophages and dendritic cells (Unanue, 1993), or activated T lymphocytes (Pichler and Coray, 1994). The expression of these molecules is regulated by such cytokines as gamma-Interferon and TNF (Kvale et al., 1988). The fact that these molecules are expressed in fetal and newborn's lymphocytes suggests that these cells are stimulated by such cytokines.

No organized lymphoid structure was seen at birth and only outlines of germinal centers were observed. Organized structures were only observed after few weeks in gut and bronchi. In our study, we could not quantify these structures, however, some authors reported that such structures number increase with time during fetal life and moreover in childhood before decreasing with age (Cornes, 1965).

Secretory component was found expressed brightly very soon after birth (2 min) by epithelial cells. This glycoprotein of 80 kD is synthesized by the basolateral plasma membrane of epithelial cells, transported through the cytoplasm, and exocytosed at the apical pole. It plays the role of IgM and dimeric IgA immunoglobulins receptor to form secretory immunoglobulins that can resist to proteolysis in secretions (Kerr, 1990). The fact that this molecule was found brightly expressed in epithelial cells suggests that (1) the mechanism of regulation of SC expression is independent from the presence of plasma cells, (2) there is no deficiency in the expression of SC in SIDS as affirmed by Ogra and colleagues (1975). This high SC expression indicates in contrast a state of stimulation in these samples. This finding agrees with the expression of class II molecules on lymphocytes mentioned before.

In conclusion, our study demonstrates that MALT maturation takes several weeks, with a small delay for isotype switching and the probable colonization of bronchial structures by cells originating from the GALT.

### MATERIALS AND METHODS

### Patients

This study involved 87 infants (58 boys and 29 girls) who died suddenly. They were aged between 2 weeks and 90 months (mean 14.7 weeks) and were forwarded to the children hospital of CHU Nancy-Brabois for systematic autopsy. Results of the autopsies and clinical investigations concluded to SIDS in 80 cases, to an infection in 2 cases, and to defined causes in 5 cases (cerebrovascular hemorrhage, acute autoimmune hemolytic anemia, Fallot's tetralogy, malignant hyperthermia, septic shock). Three children who died in postpartum respectively after 2 min, 12 hr, and 48 hr were also studied. They were one boy and two girls.

Biopsies of gut and bronchial tissues were collected during autopsy within 24 hr postdeath, snap-frozen in liquid nitrogen, and maintained at $-80$°C until studied.

### Methods

Serial frozen-cut sections (4 $\mu$m thick) were performed with a refrigerated microtome at $-30$°C and collected on glass slides. The first section of each sample was stained with toluidine blue for histological examination. The following were used for immunofluorescence.

FITC-conjugated rabbit anti-human immunoglobulins antisera were used for the detection of IgG-, IgA-, and IgM-bearing B cells and plasma cells (DAKO, Glostrup, Denmark). The expression of secretory component (SC) was investigated in direct immunofluorescence with an FITC-conjugated polyclonal goat antiserum to human SC (DAKO). After 30 min of incubation in a moist chamber at room temperature, the slides were washed three times in phosphate buffered saline (PBS), mounted in PBS-glycerol (7:3), and examined in UV light microscopy (BH2, Olympus, Japan). Plasma cells were enumerated on at least five fields at $\times 400$ magnification. Data were then expressed as number of cells per square millimeter.

### Statistics

Data were fed to a personal computer using Slidewrite Plus software (Carlsbad, CA) to appreciate the kinetics of plasma cells development in the gut and bronchi.

### *References*

Allen W. D. and Porter P. (1977) The relative frequencies and distribution of immunoglobulins-bearing cells in the intestinal mucosa of neonatal and weaned pigs and their significance in the development of secretory immunity. *Immunology* **32**, 819-824.

Chen D., Hoshi H., Tanaka K. and Murakami G. (1995) Postnatal development of lymphoid follicles in rat Peyer's patches, with special reference to increased follicle number. *Arch. Histol. Cytol.*, **58**: 335-343.

Cornes J. S. (1965) Number, size and distribution of Peyer's patches in the human small intestine. Part I. The development of Peyer's patches. *Gut*, **6**: 225-230.

Czerkinsky C., Prince S. J., Michalek S. M., Jackson S., Russell M. W., Moldoveanu Z., McGhee J. R. and Mestecky J. (1987) IgA antibody-producing cells in peripheral blood after antigen ingestion: Evidence for a common mucosal immune system in humans. *Proc. Natl. Acad. Sci. USA*, **84**, 2449-2453.

Edouard E. M. (1993) Immunoglobulins. In Fundamental Immunology, Paul W. E., Ed. (New York: Raven Press), pp. 315-382.

Eikelenboom P., Levenebach M. G. E., Van Den Brink H. R. and Streefkerk J. G. (1979) Development of T and B cell areas in peripheral lymphoid organs of the rat. *Anat. Rec.*, **194**: 523-538.

Friedberg S. H. and Weissman I. L. (1974) Lymphoid tissue architecture. Ontogeny of peripheral T and B cells in Mice: Evidence against Peyer's patches as the site of generation of B cells. *J. Immunol.*, **113**, 1477-1491.

Gascan H., Gauchat J. F., Aversa G., Van Vlasselaer P. and de Vries J. E. (1991) Anti-CD40 monoclonal antibodies or CD4+ T cell clones and IL-4 induce IgG4 and IgE switching in purified human B cells via different signaling pathways. *J. Immunol.*, **147**, 8-13.

Gutman G. A. and Weissman I. L. (1972) Lymphoid tissue architecture. Experimental analysis of the origin and distribution of T-cells and B-cells. *Immunology*, **23**, 465-481.

Kerr M. A. (1990) The structure and function of human IgA. *Biochem. J.*, **271**, 285-296.

Kvale D., Brandtzaeg P. and Lovhaug D. (1988) Up-regulation of the expression of secretory component and HLA molecules in a human colonic cell line by tumor necrosis factor-alpha and gamma interferon. *Scand. J. Immunol.*, **28**, 351-357.

Mestecky J. and McGhee J. R. (1987) Immunoglobulin A (IgA): Molecular and cellular interactions involved in IgA biosynthesis and immune response. *Adv. Immunol.*, **40**: 153-245.

Ogra P. L., Ogra S. S. and Coppola P. R. (1975) Secretory component and sudden-infant-death syndrome. *Lancet*, **2**: 387-390.

Pabst R. (1992) Is BALT a major component of the human lung immune system? *Immunol. Today*, **13**: 119-122.

Perkkio M. and Savilahti E. (1980) Time appearance of immunoglobulin-containing cells in the mucosa of the neonatal intestine. *Pediatr. Res.*, **14**, 953-955.

Pichler W. J. and Coray T. W. (1994) T cells as antigen-presenting cells. *Immunol. Today*, **15**, 312-315.

Picker L. J. and Siegelman M. H. (1993) Lymphoid tissues and organs. In Fundamental Immunology, Paul W. E., Ed. (New York: Raven Press), pp. 145-198.

Proust B., Tayot J., Malartic H. and Coqurel A. (1992) Le nourrisson décédé subitement. *Rev. Prat.*, **42**, 1743-1745.

Savilahti E. (1972) Immunoglobulin-containing cells in the intestinal mucosa and immunoglobulins in the intestinal juice in children. *Clin. Exp. Immunol.*, **11**, 415-425.

Sminia T., Janse E. M. and Plesch B. E. C. (1983) Ontogeny of Peyer's patches of the rat. *Anat. Rec.*, **207**, 309-316.

Snapper C. M. and Mond J. J. (1993) Towards a comprehensive view of immunoglobulin class switching. *Immunol. Today*, **14**, 15-17.

Spencer J., MacDonald T. T., Teresa F. and Isaacson P. G. (1986) The development of gut associated lymphoid tissue in the terminal ileum of fetal human intestine. *Clin. Exp. Immunol.*, **64**, 536-543.

Spencer J. and MacDonald T. T. (1990) Ontogeny of human mucosal immunity. In Ontogeny of the Immune System of the Gut, MacDonald T. T., Eds. (Boca Raton, FL: CRC Press), pp. 23-50.

Taubman M. A. and Smith D. J. (1993) Significance of salivary antibody in dental diseases. In Saliva as a Diagnostic Fluid, Malamud D., and Tabak L., Eds. (New York: Annals of the New York Academy of Sciences), **694**, pp. 202-215.

Torleiv O. R., Thrane P. S., Stolenberg L., Vege A. and Brandtzaeg P. (1992) Development of intestinal mucosal immunity in fetal life and the first postnatal months. *Pediatr. Res.*, **32**, 145-149.

Unanue E. R. (1993) Macrophages, antigen-presenting cells, and the phenomena of antigen handling and presentation. In Fundamental Immunology, Paul W. E., Ed. (New York: Raven Press), pp. 111-144.

Yednock T. A. and Rosen S. D. (1989) Lymphocyte homing. *Adv. Immunol.*, **44**, 313-378.

